# Supplementary material for: Rac1 Temporarily Suppresses Fertilization Envelope Formation Immediately After 1-Methyladenine Stimulation
Source: Cells. 2025 Mar 10;14(6):405. doi: 10.3390/cells14060405 (PMC11941512; doi:10.3390/cells14060405)
Supplement: Supplementary file 1 [file cells-14-00405-s001.zip › Fig S1 revised.pdf]

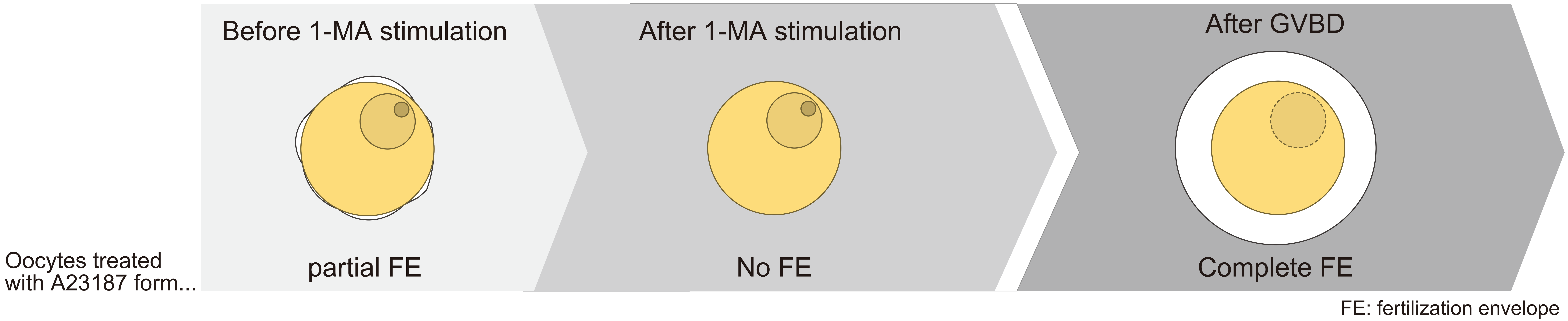
Before 1-MA stimulation
After 1-MA stimulation
After GVBD
Oocytes treated
with A23187 form...
partial FE
No FE
Complete FE
FE: fertilization envelope
