# Supplementary figures and images for: Rac1 Temporarily Suppresses Fertilization Envelope Formation Immediately After 1-Methyladenine Stimulation
Source: Cells. 2025 Mar 10;14(6):405. doi: 10.3390/cells14060405 (PMC11941512; doi:10.3390/cells14060405)

A

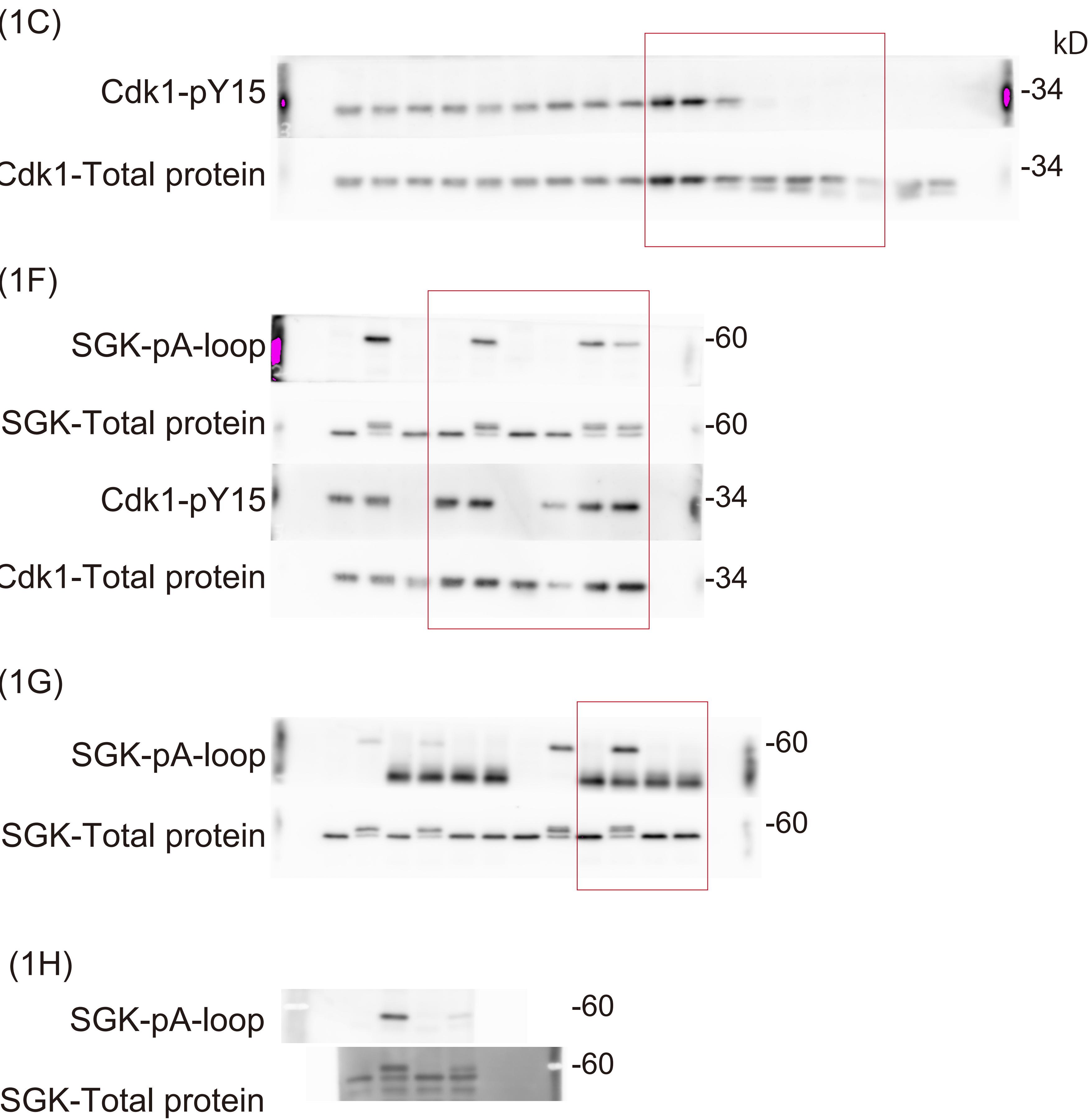

B

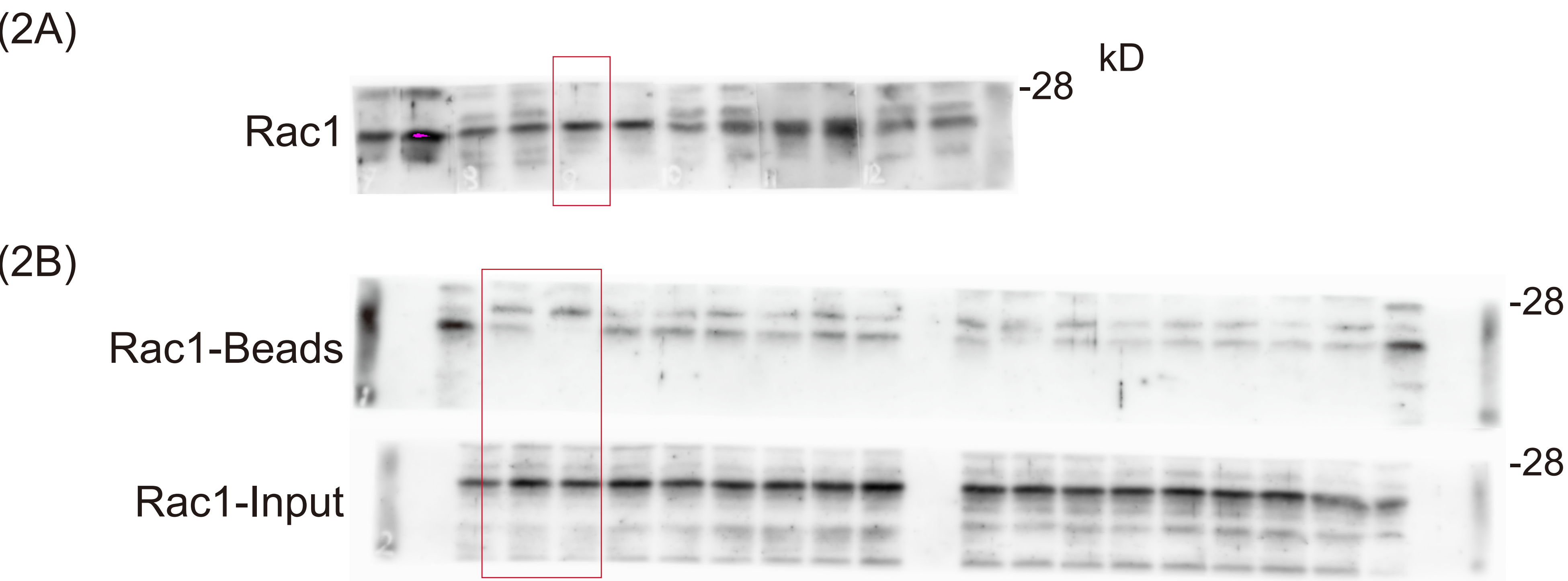

D

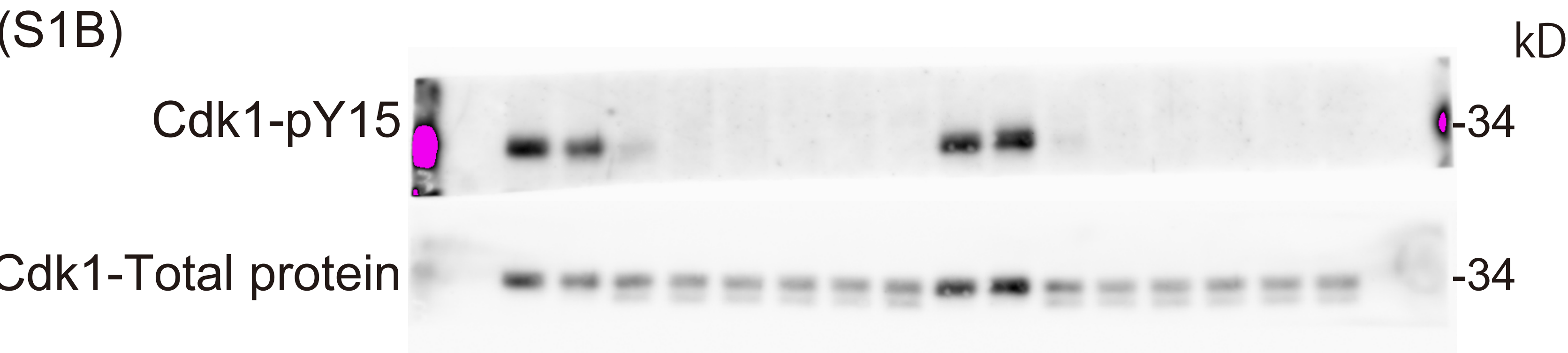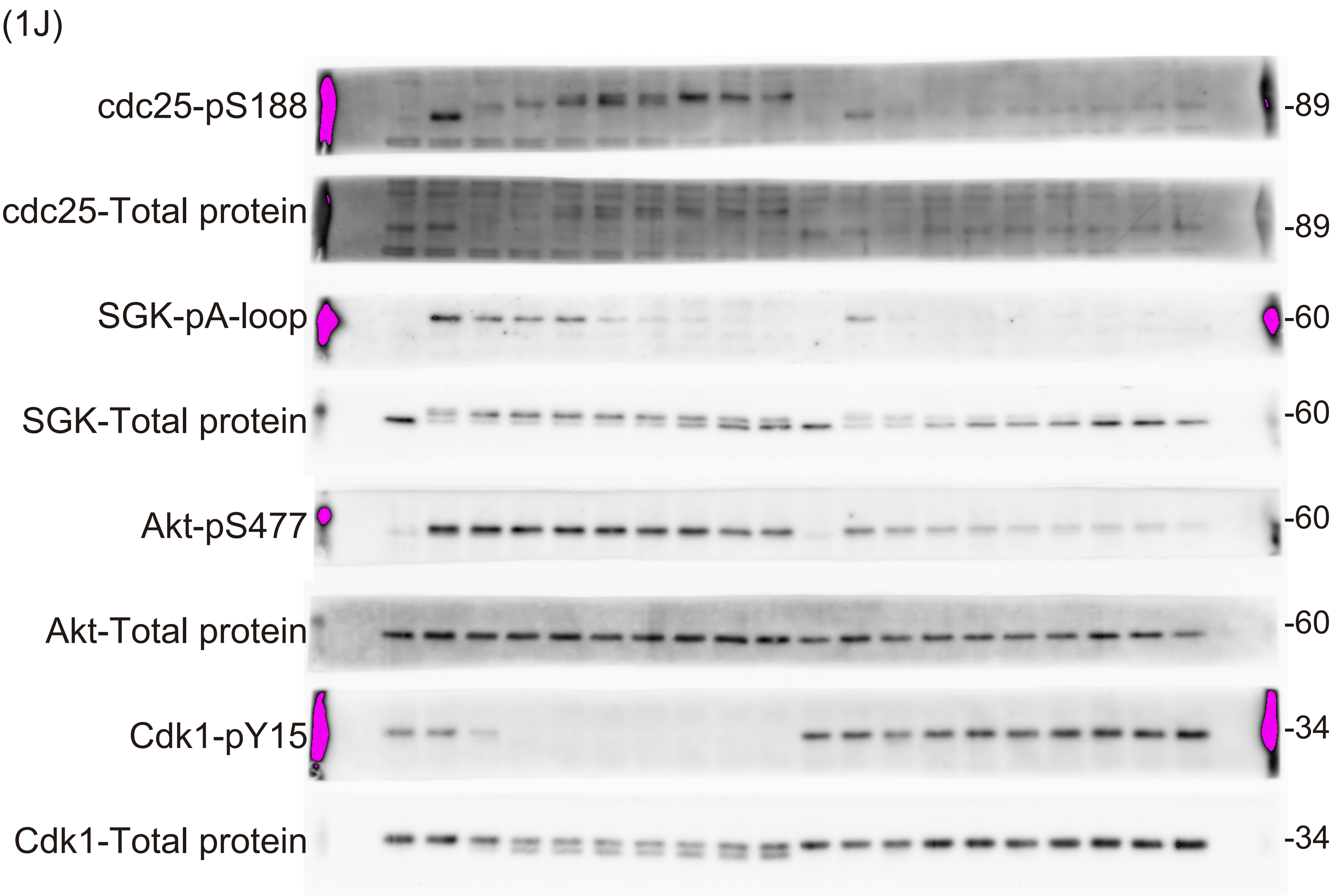

C

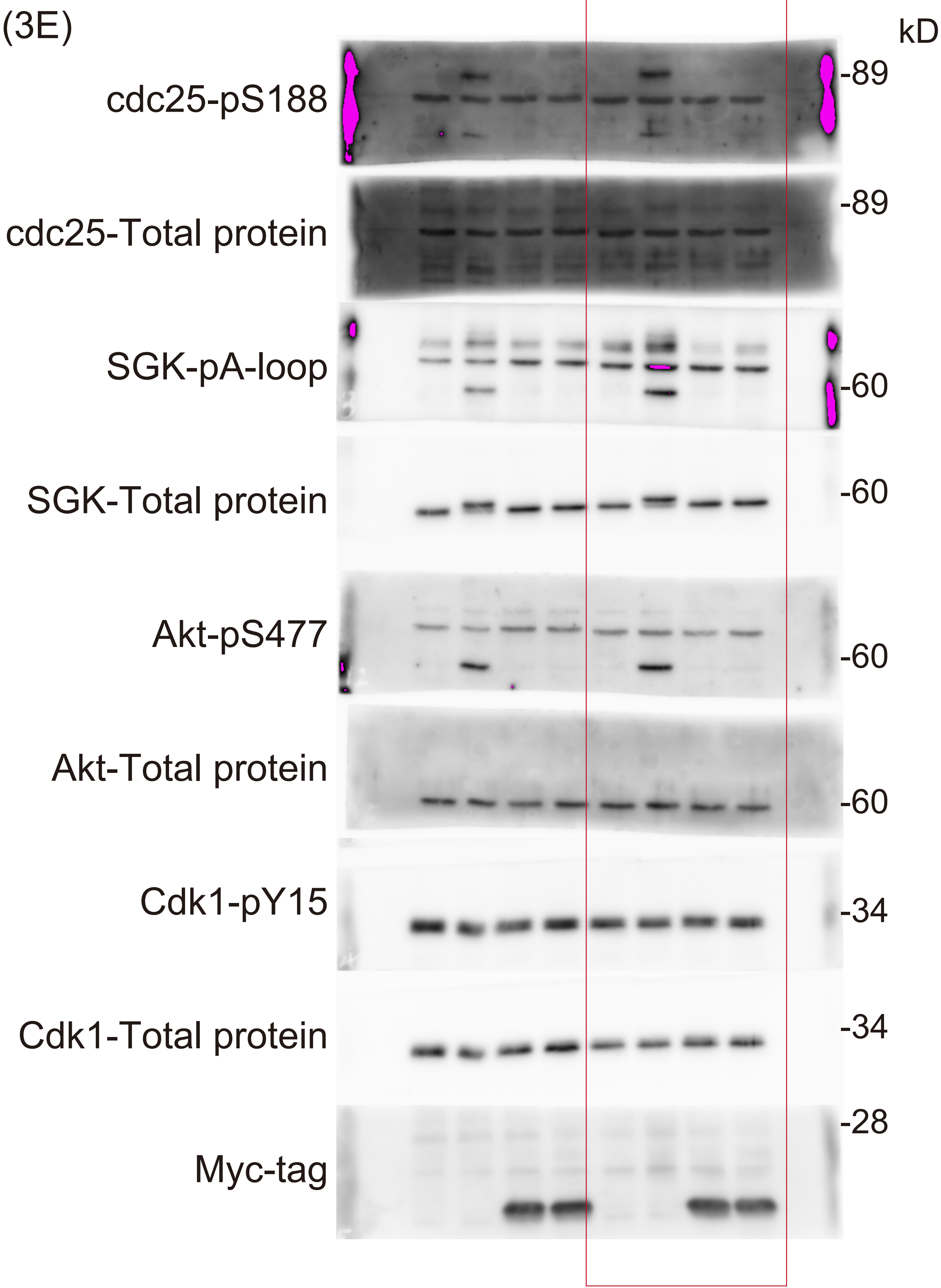

Supplement: Supplementary file 1 [file cells-14-00405-s001.zip › FigS3 revised.pdf]

**A**

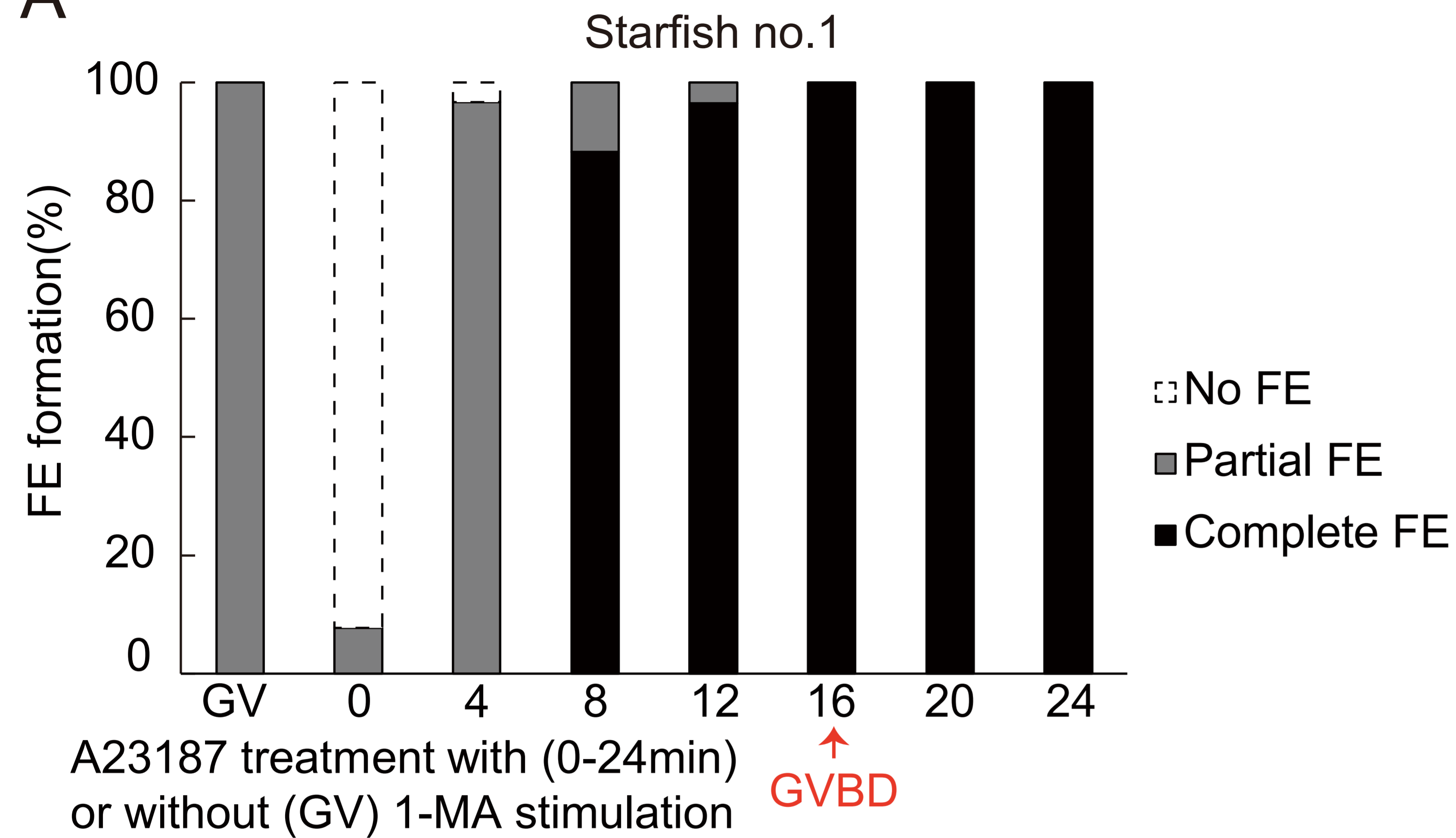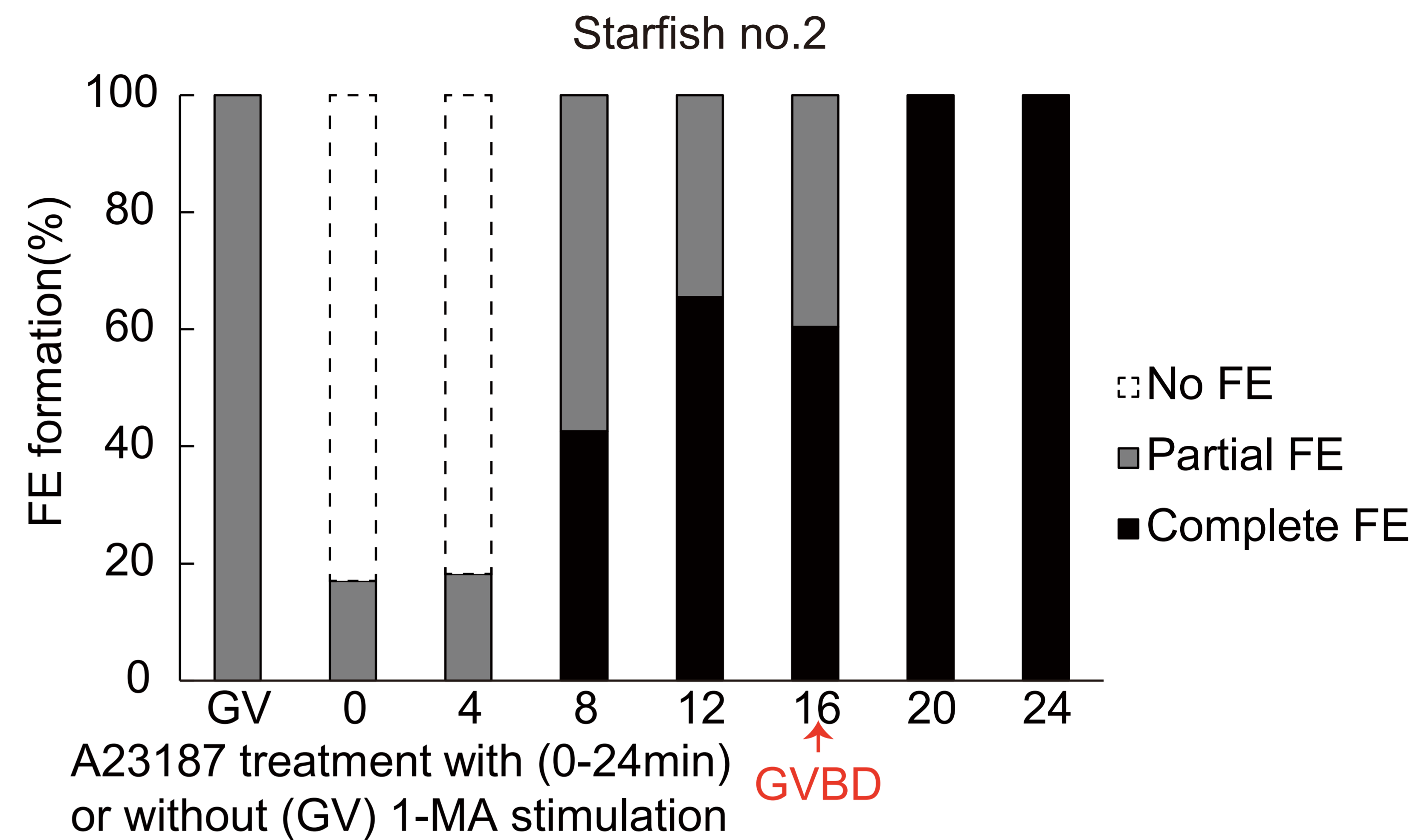

**B**

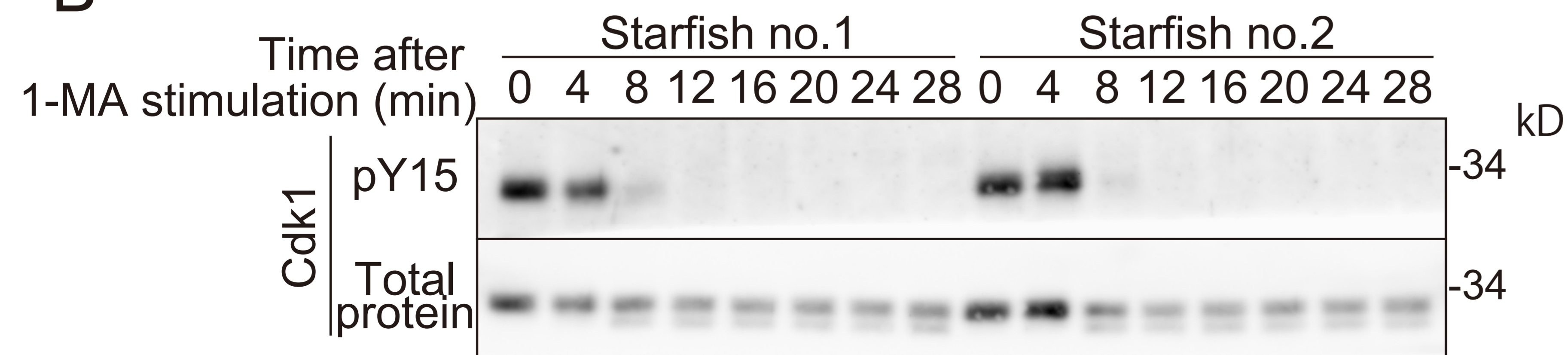

Supplement: Supplementary file 1 [file cells-14-00405-s001.zip › FigS2 revised.pdf]
